# Psammoma bodies in thyroid: are they always indicative of malignancy? A multi-institutional study

**DOI:** 10.1007/s00428-024-03934-1

**Published:** 2024-09-30

**Authors:** Esther Diana Rossi, Shipra Agarwal, Suna Erkilic, Jen-Fan Hang, Jalal B. Jalaly, Elham Khanafshar, Alexander Ladenheim, Zubair Baloch

**Affiliations:** 1 Division of Anatomic Pathology and Histology, Fondazione Policlinico Universitario “Agostino Gemelli”-IRCCS, Università Cattolica del Sacro Cuore, Largo Francesco Vito, 1, 00168 Rome, Italy; 2 Department of Pathology, All India Institute of Medical Sciences, New Delhi, India; 3 Department of Pathology, Faculty of Medicine, Gaziantep University, Gaziantep, Turkey; 4 Department of Pathology and Laboratory Medicine, Taipei Veterans General Hospital, Taipei, Taiwan; 5 Department of Pathology, Hospital of the University of Pennsylvania, Philadelphia, PA USA; 6 Department of Pathology, University of California San Francisco, San Francisco, CA USA; 7 Department of Pathology, Yale School of Medicine, New Haven, CT USA

**Keywords:** Psammoma bodies, Thyroid carcinoma, Papillary thyroid carcinoma, Thyroid follicular nodular disease, Follicular adenomas, Hashimoto thyroiditis, Lobectomy, Total thyroidectomy

## Abstract

Traditionally, psammoma bodies (PB) have been considered as tale-tell evidence of papillary thyroid carcinoma (PTC) and are frequently encountered in classic and other subtypes of PTCs. However, the presence of PBs in the thyroid gland does not always indicate malignancy. The leading hypothesis on their origin suggests that PB are remnants of papillary structures that have undergone thrombosis, necrosis, and subsequent calcification. From January 2010 to May 2024, 26 patients with psammoma bodies associated with benign thyroid lesions, mainly thyroid follicular nodular disease (TFND), Hashimoto thyroiditis (HT), Graves’ disease, and follicular adenomas, were found. The case cohort included 16 females and 10 males with a median age of 49.3 years. The series included 12 TFND, two HT, and 12 follicular adenomas (11 out of 12 were oncocytic adenomas). Twenty-four out of 26 underwent total thyroidectomy. In 24 out of 26 cases, the entire lobes and parenchyma were included and serial cuts at multiple levels were performed in cases with PB but without any evidence of malignancy. Even though the detection of PB is associated with a malignant thyroid lesion, especially PTC and its subtypes, our multi-institutional series showed that in a minority PB can be found in a variety of benign thyroid lesions. Evaluation of the entire thyroid parenchyma at multiple levels is mandatory to exclude sub-centimeter papillary thyroid carcinoma.

## Introduction

It is well-known that psammoma bodies (PB) are a common, though not entirely specific feature of papillary thyroid carcinoma (PTC) [1–7].

Morphologically, PB are usually round to oval in-shape, with lamellated calcifications; their origin and composition are debated in the literature, discriminating them from other forms of calcifications such as dystrophic calcification [1–3]. The correct interpretation of PB is based on specific morphologic characteristics including as follows: [1] round to spherical shape; [2] concentric layers of calcium deposition leading to lamellation; [3] not confined to the lumen of a thyroid follicle. It is not uncommon to find calcified and lamellated colloid encountered in follicular adenomas (FA) and carcinomas, including oncocytic type [3]. The acceptable description of PB involves the layering of calcium centered on a small group of necrotic cells, most frequently tumor cells at the tip of a papillary structure due to vascular thrombotic effects. The precise mechanism leading to the formation of PB remains unknown, and a possible immune response has been suggested, ascribed mostly to the frequent association of PB with multinucleated giant cells [8]. In the context of thyroid disease, the majority of PBs are associated with a malignant neoplasm, specifically PTC and its subtypes. They are most numerous in diffuse sclerosing subtype, PTC with *RET* rearrangements and *BRAF-*V600E mutations while less commonly in the follicular patterned *RAS*-like tumors [1–5]. It has been shown that PB are not specific to PTC and can be encountered in benign lesions such as thyroid follicular nodular disease with papillary hyperplasia, Hashimoto thyroiditis (HT), FA, including the oncocytic type, and other rare entities [9–11]. Encountering PBs in benign thyroid lesions can pose diagnostic challenges in both cytology and surgical pathology specimens [12].

The current multi-institutional study analyzes a series of cases discussing the association of PB with benign thyroid lesions.

## Material and methods

The case cohort includes contributions from five different institutions including Europe (Italy and Turkey), USA, India, and Taiwan. The surgical pathology records of the these insitutions were searched for all thyroid specimens in which the term “psammoma body” had been associated with non-malignant conditions. We performed a retrospective search over a 14-year period (from January 2010 to April 2024). The following data points were collected: age, gender, FNAC diagnoses, and surgical pathology follow-up. All available pathology slides were reviewed. Each institution has received internal institutional ethical approval for this study, with the combined approval from the Catholic University as the leading center for this study.

## Histopathology specimens

The processing of all surgical pathology specimens was standard and based on fixation in 10% buffered formaldehyde, embedding in paraffin, sectioning into 5-micron-thick slides, and then staining with hematoxylin–eosin (H&E). The diagnoses were classified according to the 5th WHO Classification of Tumors of Endocrine Organs [13]. The follow-up period ranged from 1 to 120 months. The cases with PB in the parenchyma were recut at multiple levels, as well as some of the adenomas in few of the institutions (Europe, Italy mostly).

The number of blocks per cases ranged between 4 and 12, with a number of blocks with PBs ranging from 1 to 7. Considering the quantity of PBs, in each of our cases, we found some to numerous PBs. None of our cases showed a single PB.

## Results

A total of 26 cases of thyroid specimens diagnosed with the presence of PB associated with non-malignant thyroid lesions are included in this study. The patient demographics included 10 men and 16 women with a median age of 49.3 years. Twenty-four (92.3%) out of 26 underwent total thyroidectomy and follow-up evaluation is available for all, ranging from 1 to 120 months.

**Fig. 1:**
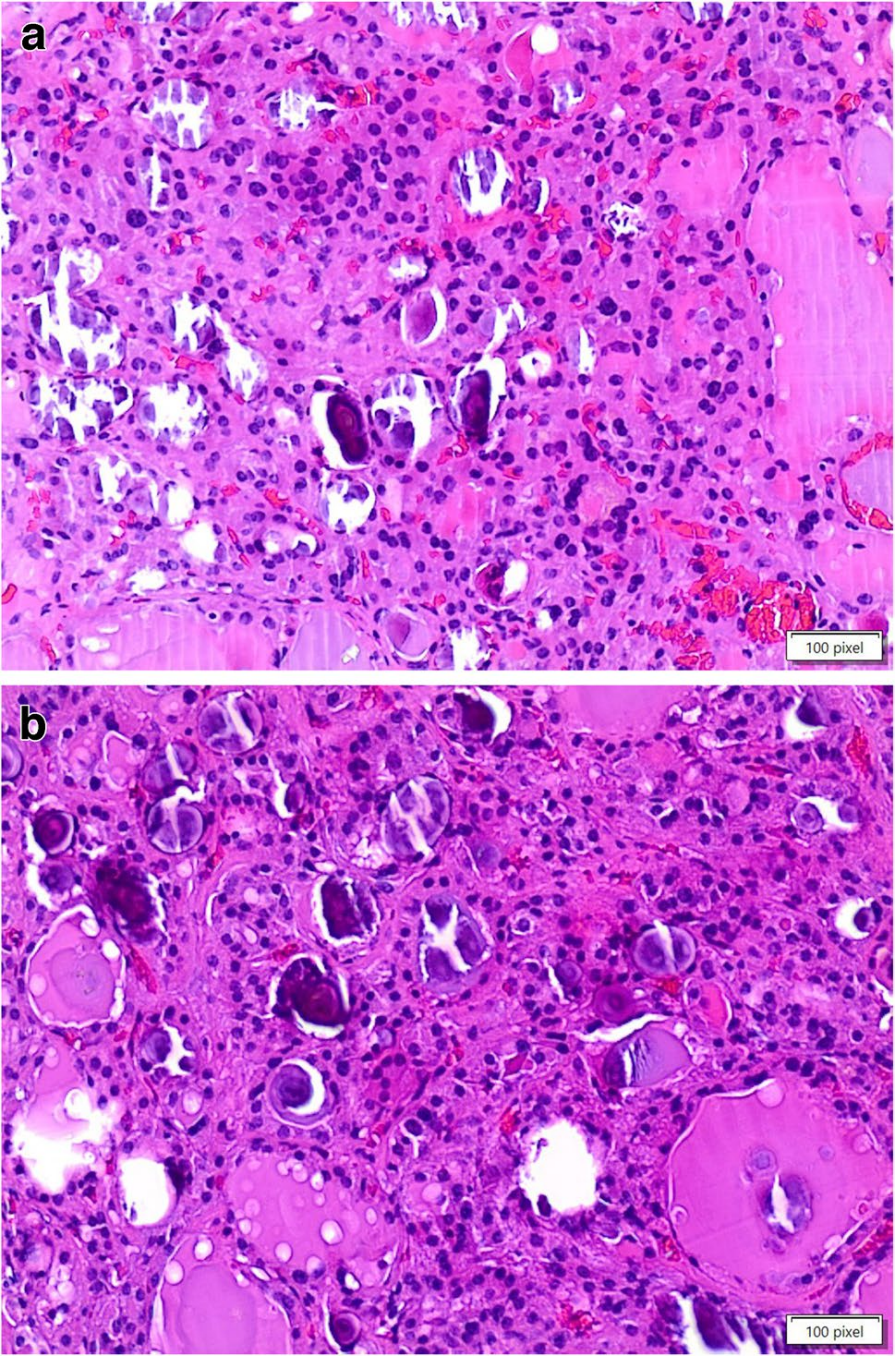
**A**, **B** The pictures show an oncocytic adenoma with evidence and details of psammoma bodies within the lesion (H&E, 200 ×)

**Fig. 2:**
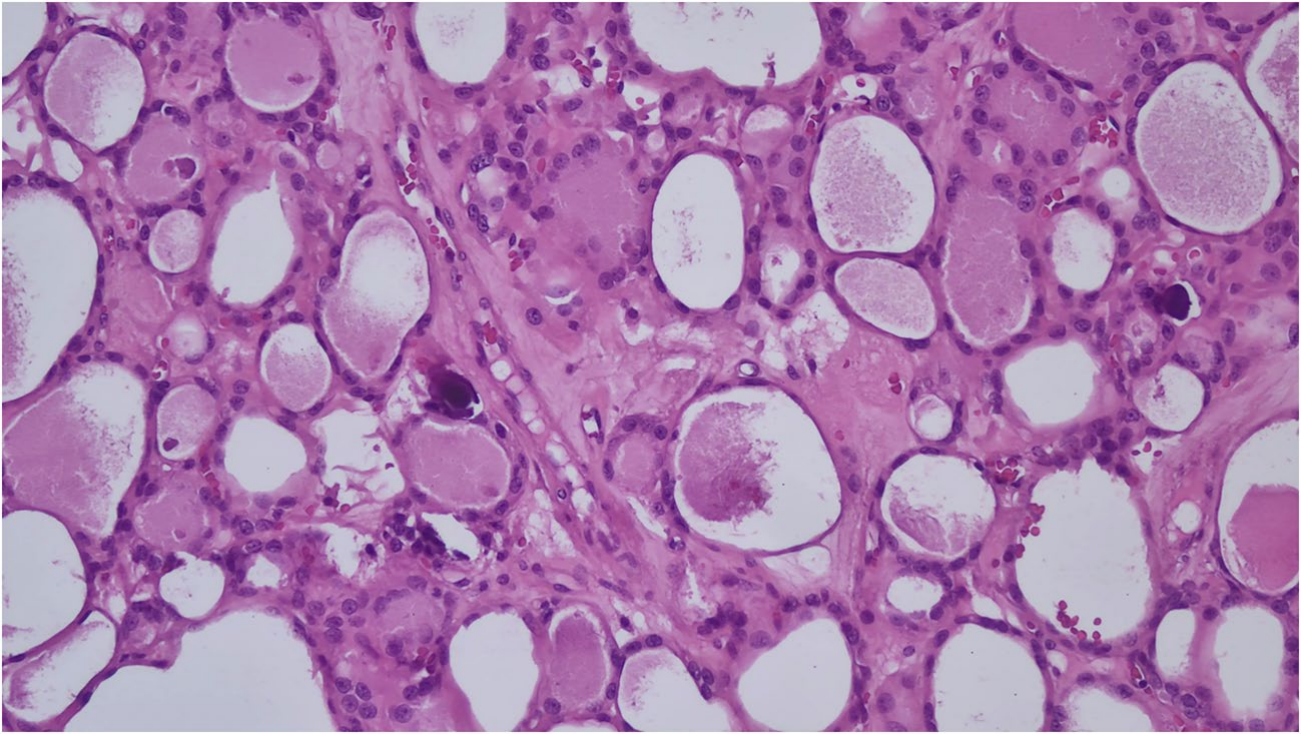
The picture shows the evidence of psammoma bodies in the contest of a thyroid follicular nodular disease (H&E, 200 ×)

**Fig. 3:**
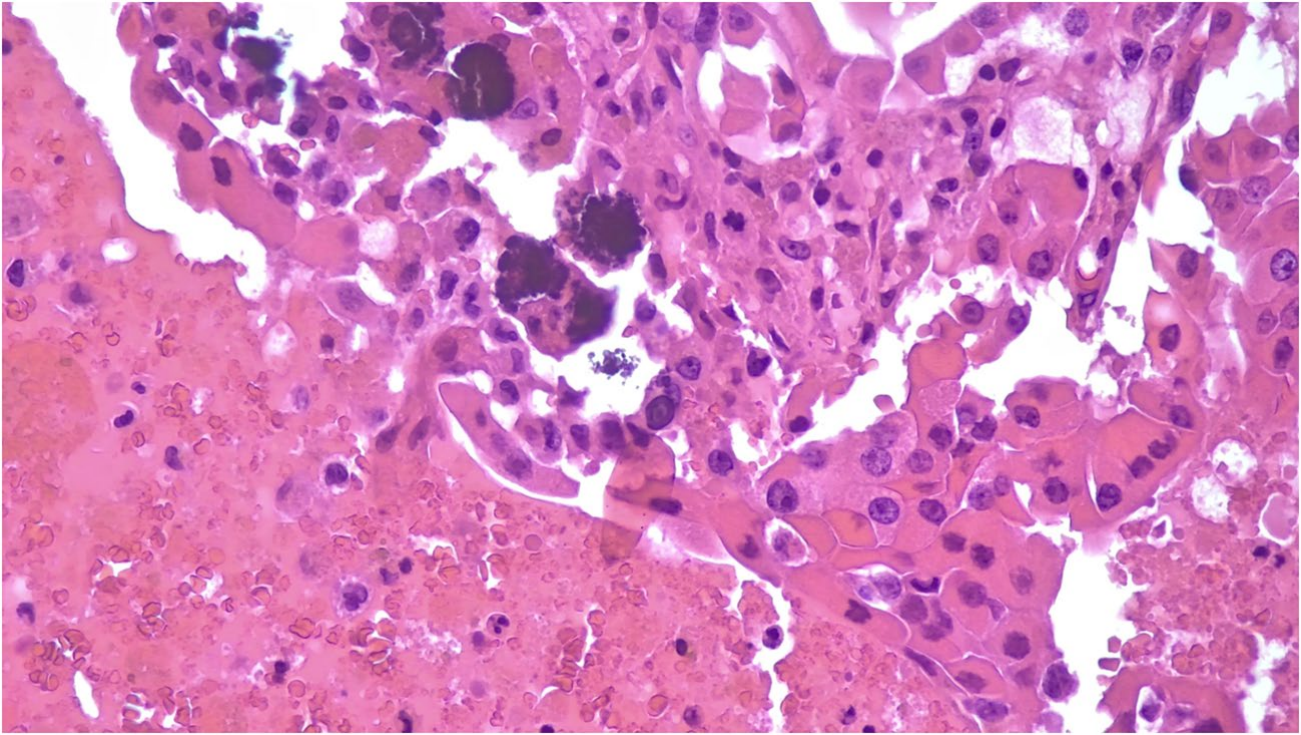
The picture shows the details of psammoma bodies in the contest of oncocytic cells in a case diagnosed as oncocytic adenoma (H&E, 400 ×)

The diagnoses included 12 adenomas (one follicular and 11 oncocytic adenomas, Fig. 1A–B and Fig. 3), two chronic lymphocytic thyroiditis (HT) including one case with extensive oncocytic metaplasia, and 12 cases of thyroid follicular nodular disease (TFND) (Table 1, Fig. 2). In 24 of 26 cases, the thyroid parenchyma was entirely included at various levels, to find sub-centimeter PTC which had been missed in the first evaluation (Table 2). In the remaining two cases (one oncocytic adenoma, one follicular nodular disease), the nodules with psammoma bodies were entirely embedded (Fig. 3). None was found to harbor carcinoma after detailed sectioning. None of the patients have shown any evidence of thyroid carcinoma on follow-up.

**Table 1:** Details about the cases from the different institutions

	Age (years (range))	Size (cm (range))	Gender (No.)	Histologic diagnosis (No.)	Follow-up
Europe (*n* = 14)	23–75	1.4–4.5	F = 9 M = 5	TFND with PB = 5 OA = 6 FA = 1 HT = 2	NED
India (*n* = 1)	43	Lack of nodular lesion	M = 1	TFND with PB = 1	NEB
Taiwan (*n* = 3)	64–78	1.3–6.1	F = 2 M = 1	TFND with PB = 2 OA = 1	NED
USA (*n* = 8)	26–69	0.4–2.8	F = 5 M = 3	TFNB with PB = 4 OA = 4	NED
Total (26 cases)					

Legend: *M*, male; *F*, female; *FA*, follicular adenoma; *OA*, oncocytic adenoma; *PB*, psammoma bodies; *TFND*, thyroid follicular nodular disease; *NED*, no evidence of disease

**Table 2:** Summary of the distribution of psammoma bodies across the various benign diagnoses

	F/M	TFND	HT	FA/OA	TT/lobect
Cases	16/10	12	2	1/11	24/2

The PBs were found in the thyroid parenchyma in case of thyroid follicular nodular disease and thyroiditis, and within the adenomas, particularly oncocytic adenomas. Specifically, in the cases with chronic lymphocytic thyroiditis, PBs were seen close to the foci of oncocytic metaplasia.

## Discussion

Psammoma bodies not associated with a malignant thyroid lesion can pose diagnostic conundrums, as it represents one of the diagnostic features of PTC and its subtypes. In a paper by Bai et al., the authors identified PB in 25% (58 out of 229 cases) of PTCs and its statistically significant correlation with lymph node metastases (*p* = 0.0347) and high stage, especially stage IVa (*p* = 0.0255), and extrathyroidal extension (*p* = 0.0618) [6]. Consorti et al. studied a series of 196 patients, including 33 cases of differentiated thyroid carcinoma, nine FA, and 154 thyroid follicular nodular disease, demonstrating that PB and calcifications are more commonly seen in differentiated thyroid carcinoma compared to benign lesions (34% versus 31.2% of global benign lesions) [12]. However, this latter study also supports the notion that PB can be encountered in benign thyroid lesions with some frequency [12].

The exact origin and significance of PB in benign cases remain unclear and under evaluation [1–5]. It is commonly agreed-upon that PBs represent infarcted and necrotic cells from papillary structures of a PTC which have been layered in a concentric fashion with calcific deposits. This is further confirmed by the published data which has clearly demonstrated that PB are a feature for a PTC, including those cases in which cancer is not readily identified [2–6]. The question arises for those rare scenarios in which, despite all efforts (submission of entire thyroid resection, deeper levels), a neoplasm is not encountered. This diagnostic dilemma has been tackled by studies which have documented that PBs can be seen in benign thyroid lesions.

Hunt et al. studied 29 cases with psammomatous calcification in the absence of any malignancy [1]. Nonetheless, the search for any carcinoma, with the inclusion of the entire thyroid tissue, documented that 27 out of 29 [1] cases had an ipsilateral or contralateral thyroid carcinoma, including 15 cases with a larger than 1.0-cm neoplasm and 12 with a < 1.0 cm-sized PTC. Only two cases showed the presence of PB without any malignant component; however, both patients underwent a lobectomy, and the entire specimen was not submitted for histopathologic evaluation, raising the possibility that an occult PTC may have been missed in these cases [1]. As evidenced by this study, there is a strong association between PBs and PTC, and having these in a benign thyroid lesion was a rare occurrence. The current multi-institutional series adds to the existing evidence that PB can occur in benign conditions, such as thyroid follicular nodular disease, HT, Graves’ disease, and FA. Interestingly, most cases of adenoma with PB were oncocytic type. We do not have a specific reason for the association; Montone et al. proposed that modified colloid, produced by the oncocytic cells, is likely to attract calcium, leading to the formation of psammoma bodies [13]. Other possibilities include the well-documented tendency to infarction in oncocytic neoplasms, either due to abnormal angiogenesis [14] or due to metabolic instability in oncocytic cells.

Taking into account that likelihood of having a PTC in the presence of PBs is high, reporting benign lesions with PBs can be challenging for pathologists as well clinicians managing patients. Should all patients with psammoma bodies associated with benign lesions be considered as harbinger of malignancy (intralymphatic spread of a vanishing PTC) and treated as such or this occurrence be considered as in an otherwise benign lesion be considered incidental finding?

The current series, including cases from different institutions, showed that the evidence of PB is likely to be associated to different benign thyroid conditions, including TFND, HT, and follicular adenomas, mostly oncocytic adenomas. The majority of our cases had a total thyroidectomy (92.3%), showing the lack of any focus of thyroid carcinoma. The data from our series confirmed the other series from literature and the accuracy in including the entire thyroidal tissue with multiple levels of cut.

As it stands for now, there is no unified answer how to handle the presence of PBs without any malignant focus. The burden of proof lies on the clinical assessment, and as noted by Hunt et al., PB are more commonly associated with the detection of ipsilateral or contralateral malignant foci than being associated with a benign pathology [1]. The first suggestion is to analyze multiple levels of the thyroid lobe and parenchyma to exclude a small focus of PTC. It will not be farfetched to recommend that, during the search for a small sized focus of PTC, the entire thyroid gland specimen should be submitted for histopathologic interpretation. Furthermore, the case may necessitate several deeper levels of the block to hunt for a possible missed neoplastic focus. Nonetheless, in a minority of cases, even after having done all these steps, a carcinoma maybe not found, confirming that PB can be associated with benign conditions in small number of cases as evidenced by prior reports and the current multi-institutional study. A review article by Triggiani et al. documented that PB have been found in 5% of benign thyroid lesions, including goiters, lymphocytic thyroiditis, and adenomas [9]. Nonetheless, other authors such as Ferreira et al. discussed the nature of PB and the importance of discrimating PB from other types of microcalcifications and macrocalcifications in the thyroid parenchyma, emphasizing that PB are composed of a peculiar concentric lamellation, lacking birefringence [3].

It is well-known that small calcifications can be encountered in the ultrasound evaluation of thyroid nodule for FNA and are often termed as “punctate echogenic foci” which may correlate to psammomatous calcifications (Alexander et al. [15]). As reported by O’Connor et al., an accurate ultrasound evaluation is likely to find a strong and significant association between the presence of microcalcifications and PB on histologic samples of thyroid malignancy (*p* < 0.001) [5]. Nevertheless, in contrast to PBs, the detection of macrocalcifications shows a different morphological appearance and it is frequently found in benign diseases or as a post-aspiration effect. O’Connor et al. documented that in their cohort of 71 benign lesions, only 7% had microcalcifications and none of them had PB [5]. As a further evidence, Mohammed et al. also found the presence of PB, diffusely distributed within a lesion characterized by bundles of spindle muscle cells with blunt-ended nuclei suggestive for a thyroid leiomyoma [9]. For this patient too, the entire thyroid tissue was submitted for evaluation without detecting any microscopic carcinoma.

Compared to histologic specimens, the predictive role of PBs for malignancy in fine needle aspiration (FNA) samples lacking other diagnostic features of PTC can be more challenging. As per the Bethesda System for Reporting Thyroid Cytology, presence of psammoma bodies in an aspirate with cellular atypia should be classified as “Atypical” [16]. Ellison et al. reported that PB were found in eight out of 313 thyroid FNA. However, the authors did not report specific figures about the number of cases, if the thyroid glands were entirely embedded. For this reason, their results, showing their positive predictive value for PTC was only 50%, should be evaluated in the contest of the bias of their data. Their results suggested that PBs could be associated with benign lesions and represent an unreliable feature of PTC [7].

In conclusion, this study documents the presence of PBs in a set of 26 benign thyroid lesions. However, since these are considered as one of the diagnostic features of PTC, every effort should be made to analyze the thyroid specimen in its entirely to exclude the possibility of a small focus of PTC undetected by gross pathologic examination [14, 15].

## Data Availability

There is a word file including the details of our cases.

## Declarations

Ethical approval from the Internal Committee Policlinico Gemelli 2023 *n** 123.
